# Pretreatment of Nicorandil Protects the Heart from Exhaustive Exercise-Induced Myocardial Injury in Rats

**DOI:** 10.1155/2022/7550872

**Published:** 2022-01-06

**Authors:** Lei Lv, Lin Li, Yiyong Zhu, Anwar Azhar, Yao Li, Yongyuan Wang, Tao Jin, Xin Yin, Xi Chen, Yu Liu, Yong Zhong

**Affiliations:** ^1^Department of Geriatric Cardiology, Jinling Hospital, Nanjing, Jiangsu 210002, China; ^2^Health Care Office, Jinling Hospital, Nanjing, Jiangsu 210002, China; ^3^School of Life Sciences, Nanjing University, Nanjing, Jiangsu 210093, China; ^4^Department of Health Medicine, Jinling Hospital, Nanjing, Jiangsu 210002, China; ^5^Department of Geriatrics, Jinling Hospital, Nanjing, Jiangsu 210002, China

## Abstract

**Objective:**

Nicorandil has been widely used for the treatment of angina pectoris and myocardial infarction. The purpose of this study was to investigate whether nicorandil plays a protective role in exhaustive exercise (EE)-induced myocardial injury.

**Methods:**

Here, we applied the rat EE model and treated them with exercise preconditioning (EP, reported to protect the heart) or different doses of nicorandil gavage, respectively, to explore whether there are protective effects of single EP or nicorandil or a combination of both and the potential mechanism. Forty-nine male Sprague Dawley rats were randomly divided into control, EE, EP + EE, nicorandil (with low, middle, and high dose) + EE, and EP + nicorandil (middle dose) + EE. Blood samples and myocardial tissues were collected to analyze the myocardial injury-related index.

**Results:**

EE induced myocardial structural damage and altered the myocardial injury markers, which were partially reversed by pretreatment of nicorandil. In addition, oxidative stress and inflammation lead to the accumulation of reactive oxygen species (ROS) products and further damage to the myocardium, while pretreatment of nicorandil reduces the oxidative stress response and inflammation. Moreover, nicorandil suppressed the myocardial apoptosis induced by EE, as indicated by a decrease of Bax and caspase-3 expression and an increase of Bcl-2 expression. Finally, the pathway in which nicorandil plays a role may be involved in the endothelial nitric oxide synthase (eNOS)/nitric oxide (NO) pathway. Pretreatment of nicorandil increased the protein level of myocardial eNOS and NO production.

**Conclusion:**

Our result demonstrated that nicorandil has protective effects in EE-induced myocardial injury with dose-dependent effects. A combination of nicorandil and EP can further improve the protective effects. Taken together, nicorandil can be potentially used as an intervention method in EE-induced myocardial injury.

## 1. Introduction

Numerous research studies have well documented the benefits of exercise, such as improved maximal oxygen consumption, weight loss, skeletal muscle function, myocardial health, and so on. However, acute exhaustive exercise (EE) was reported to induce sustained myocardial ischemia and hypoxia, which is beyond the body's bearing limit, and finally, induce myocardial injury [1–3]. Exercise can protect the heart from myocardial injury by activating several pathways such as phosphatidylinositol 3-phosphate kinase (PI3K)/protein kinase B (AKT)/mammalian target of rapamycin (mTOR), EGFR/JNK/SP-1, and nitric oxide (NO)-signaling [4]. Recent studies have well documented the protective effects of exercise preconditioning (EP) in EE and ischemia/reperfusion (I/R) injury by activating the pathways abovementioned [5], which initiate absolute or relative recurrent transient myocardial ischemia, and this is similar to the ischemic preconditioning (IP) process [6–8].

Intriguingly, nicorandil, which is an adenosine triphosphate-sensitive potassium (KATP) channel activator and a donor for NO and can serve valid benefits to acute myocardial infarction (AMI) [9, 10] in basic and clinical research, has not been investigated during acute EE-induced myocardial injury. Clinical research has shown that the cardioprotective effects of nicorandil are similar to the effects of IP in ischemic injury and myocardial infarction patients [10].

Vitro experiments also demonstrated the antiapoptotic effect of nicorandil mediated by mitochondrial KATP channels in cultured cardiac myocytes [11]. Animal studies further proved that nicorandil treatment reduced the size of myocardial infarction in I/R models [12]. Therefore, whether nicorandil could serve as an effective intervention method to prevent or reduce EE-induced myocardial injury needs to be studied.

Here, we used a rat model treated with EE together with EP or nicorandil to elaborate whether there are cardioprotective effects of nicorandil in EE.

## 2. Materials and Methods

### 2.1. Animal Model

Forty-nine male Sprague Dawley (SD) rats were purchased from Shanghai B&K Laboratory Animal Technology (150–170 g) (license number: SCXK (Shanghai) 2018–0006) and housed in a standard animal facility. The room temperature was 20 to 22°C with a 12/12 light-dark cycle. Rats were fed with standard rodent mash and water ad libitum. Subsequently, rats were randomly divided into 7 groups: the control group (CON; without any treatment, *n* = 7); the exhaustive exercise group (EE; trained with EE, *n* = 7); the exercise preconditioning group (EP; EE is carried out after EP, *n* = 7); the low-dose nicorandil group (NL; EE after low-dose nicorandil treatment, *n* = 7); the medium-dose nicorandil group (NM; EE after medium-dose nicorandil treatment, *n* = 7); the high-dose nicorandil group (NH; EE after high-dose nicorandil treatment, *n* = 7); and the nicorandil exercise preconditioning group (NM + EP; exercise preconditioning and EE after middle-dose nicorandil treatment, *n* = 7). All use of animals was followed by the Guide for the Care and Use of Laboratory Animals and approved by the Ethics Committee of Jinling Hospital.

### 2.2. Nicorandil Treatment

Rats in the CON and EE groups were pretreated with physiological saline solution (3 mg/kg/d), while the rats in all nicorandil groups were pretreated with different doses of nicorandil (NL: 1 mg/kg/d, NM: 3 mg/kg/d, NH: 9 mg/kg/d, and NM + EP: 3 mg/kg/d) by gavage for 4 weeks before the EE section.

### 2.3. Treadmill Experiment

Adaptive treadmill running was applied to all rats with a protocol of 10–15 m/min for 15 minutes at a slope of 0° per day for 5 days. Rats that could not run were eliminated. All exercise groups followed a warm-up protocol with an initial velocity of 10 m/min for 5 minutes and a gradual acceleration to 30 m/min within 5 minutes. The EP protocol referred to early studies [5]. In brief, rats underwent 4 cycles of 10 minutes of high-intensity running (28–30 m/min) with a 10-minute interval within each cycle, and the last cycle of running was accompanied by 10 minutes of gradual deceleration to cool down. The EE protocol referred to the article by Yuan et al. [13]. In brief, rats underwent progressive acceleration until the speed reached 30 m/min. Continuous running was maintained at 30 m/min until exhaustion [13]. Rats were sacrificed 30 minutes after exhaustion. Myocardial and blood samples were collected and stored at −80°C for analysis.

### 2.4. Blood and Myocardial Samples

Blood samples were collected from the right atrium at the end of the experiment and centrifuged at 1500 g for 15 minutes of serum separation and kept at −80°C. After blood collection, the rats' hearts were surgically excised under sterile and RNase-free conditions. The rats' left ventricular apexes were taken, quickly frozen in liquid nitrogen, and stored at −80°C.

### 2.5. Hematoxylin and Eosin (HE) Staining

The preprepared paraffin sections were deparaffinized with xylene, then treated with a gradient of ethanol, and then washed with tap water. The deparaffinized tissue section was dyed with hematoxylin dyeing solution for 5 minutes and then washed with tap water. Next, the differentiation solution was applied, and the tissue samples were washed in tap water. The samples were dehydrated with an ethanol gradient, followed by xylene transparent and sealed with neutral gum. Finally, an optical microscope was used to observe and photograph.

### 2.6. Electron Microscope

After the experiment, the rats were killed, and tissue samples of 1 mm^3^ were taken 4 mm from the apexes. The samples were fixed in 2.5% glutaraldehyde in 0.1 mol/l phosphate buffer at 4°C for 4 hours and postfixed in 1% buffered osmium tetroxide at 4°C for 2 hours. The tissue blocks were dehydrated in graded ethanol and embedded in epoxy resin. Ultrathin sections were stained with uranyl acetate and lead citrate and viewed under a Joel JEM-1011 transmission electron microscope (Joel Ltd., Japan).

### 2.7. Measurement of Myocardial Injury Biomarkers

Creatine kinase (CK), CK-MB activity, lactate dehydrogenase (LDH), and cardiac troponin I (cTnI) levels in the serum were measured by using the Hitachi Automatic Analyzer (7600, Hitachi, Japan).

### 2.8. Measurement of Inflammation and Oxidative Stress

The levels of tumor necrosis factor *α* (TNF-*α*) and interleukin 6 (IL-6) in serum were detected by using an enzyme-linked immunosorbent assay (ELISA) kit (Cusabio, Wuhan, China) in accordance with the manufacturer's instructions. Superoxide dismutase (SOD) activity and malondialdehyde (MDA) content in the serum were measured by using a commercial kit (Jian Cheng Bioengineering Institute, Nanjing, China) with spectrophotometry.

### 2.9. NO and Endothelin-1 (ET-1) Measurement

Tissue samples from left ventricular zones were washed, homogenized (1 : 10, wt⁄vol deionized water), and centrifuged at 16000 g for 10 minutes. The Bradford method (Bio-Rad) was used to measure the protein concentration. The concentration of nitrite, a stable metabolite of NO, was measured using a NO detection kit (JianCheng Bioengineering Institute, Nanjing, China) to determine the total NO production in the supernatant. The ET-1 level in the rats' hearts was measured by ELISA (Cusabio, Wuhan, China).

### 2.10. Western Blotting

Heart tissue was homogenized using a microdismembrator on ice in RIPA lysis buffer containing phosphatase and protease inhibitor cocktail (1 : 50 diluted) for 30 minutes. Lysates were centrifuged at 16000 g for 30 minutes at 4°C. The supernatant was incubated with 10% SDS buffer at 99°C for 5 minutes. The total protein content of myocardial tissue was extracted, and an equal amount of protein was electrophoresed on sodium lauryl sulfate-polyacrylamide gel (SDS-PAGE). The protein was then transferred to a PVDF membrane and blocked with 5% skimmed milk powder in TBST (pH 7.4, TBS with 0.1% Tween-20) for 1 hour. Blots were incubated with corresponding primary antidodies against cardiac troponin T (Abcam, ab209813, 1 : 1000), cardiac troponin I (Abcam, ab209809, 1 : 1000), Bax (CTS, 2722, 1 : 1000), Bcl-2 (CTS, 3498, 1 : 1000), cleaved caspase-3 (CST, 9664, 1 : 1000), and endothelial nitric oxide synthase (eNOS) (Abcam, ab76198, 1 : 1000) overnight at 4°C. The GAPDH (Abcam, ab8245, 1 : 2000) was served as the internal control. After washing, blots were incubated with suitable secondary antibodies conjugated to horseradish peroxidase. The protein bands developed with the horseradish peroxidase developer solution were quantified using chemiluminescence.

### 2.11. Statistical Analysis

The data were presented as mean ± standard error (SE) and analyzed by GraphPad Prism 7.0 software. A one-way analysis of variance (ANOVA) was used to analyze the results of each experimental group. *P* values <0.05 were considered statistically significant.

## 3. Results

### 3.1. HE Staining Findings

**Figure 1:**
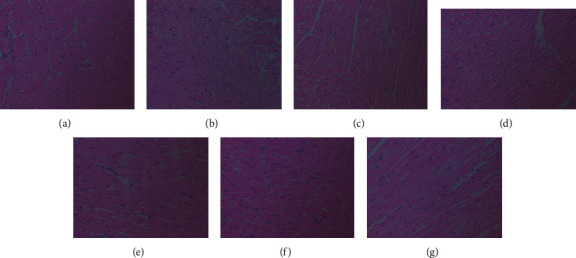
Representative photomicrographs of HE staining. Photographs had the same magnification (×200). (a) CON group, (b) EE group, (c) EP group, (d) NL group, (e) NM group, (f) NH group, and (g) NM + EP group.

The rats treated with EE showed myofibrillar loss, cardiomyocyte necrosis, and structural abnormalities, while pretreatment with different doses of nicorandil or EP or a combination of both partially reversed this change (Figure 1).

### 3.2. Electron Microscopy Findings

**Figure 2:**
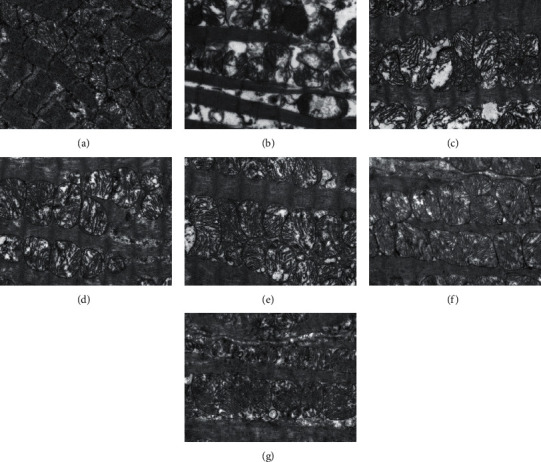
Examples of electron micrograph. In comparison with the CON group (a), the ultrastructure in the EE group (b) showed widespread mitochondrial damage, with severe disturbance in the mitochondrial crista arrangement, loss of mitochondrial matrix substance, the presence of intramitochondrial vacuoles, and disruption of the mitochondrial membrane. On the contrary, the ultrastructure of myocardial cells of EP and nicorandil pretreated rats (c–f) was well preserved with only a few cells being slightly damaged. In the NM + EP group (g), the cristae were largely intact, some cristae were flocculent, and the damage was insignificant.

In the CON group (Figure 2(a)), the ultrastructure of the myocardium was normal. Myofibrils were regularly arranged. Mitochondria had complete structure and well-developed cristae. In the EE group (Figure 2(b)), the ultrastructure of myocardial cells was damaged. Swollen mitochondria were observed with cristae becoming fuzzy or ruptured and vacuole degeneration being noted. In contrast, reduced damage to mitochondria was observed in the EP and nicorandil pretreatment groups (Figures 2(c)–2(f)). It was particularly obvious in the medium-dose and high-dose nicorandil pretreatment groups. In the NM + EP group, the cristae were largely intact, only few cristae were flocculent, and the damage was insignificant (Figure 2(g)).

### 3.3. Myocardial Injury Biomarkers

The activity of serum CK, CK-MB, and LDH significantly increased in the EE and NL groups compared with that of the CON group, while pretreatment of EP, all doses of nicorandil, and the combination of NM and EP significantly lowered the CK and LDH levels in comparison with the EE group. Pretreatment of NM, NH, and the combination of NM and EP significantly lowered the CK-MB level in comparison with that of the EE group.

**Figure 3:**
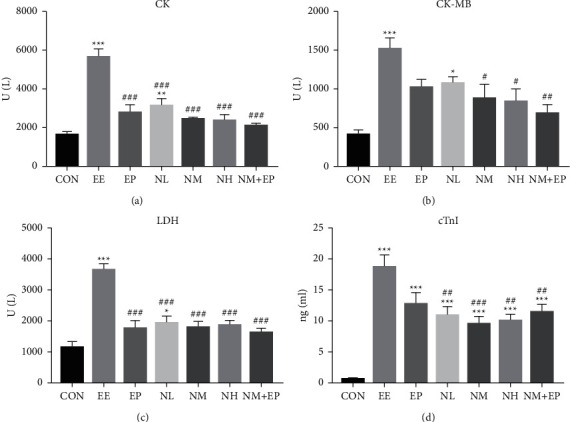
Effects on the activity of myocardial injury biomarkers (CK, CK-MB, LDH, and cTnI) in the rat serum (*n* = 7) after applying nicorandil. One-way ANOVA statistical analysis was conducted for multiple comparisons of group means. The same was used to analyze the results of each experimental group. ^*∗*^*P* < 0.05, ^*∗∗*^*P* < 0.01, and ^*∗∗∗*^*P* < 0.001 compared with the CON group; ^#^*P* < 0.05, ^##^*P* < 0.01, and ^###^*P* < 0.001 compared with the EE group.

The level of serum cTnI significantly increased in all groups compared with that of the CON group, while pretreatment of all doses of nicorandil and the combination of NM and EP significantly decreased the level of cTnI in comparison with that of the EE group (Figure 3).

### 3.4. SOD Activity and MDA Level

**Figure 4:**
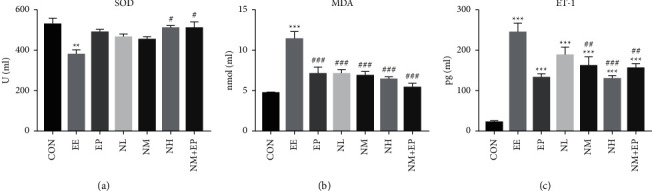
Effects on the SOD activity and MDA level in the rats' serum and ET-1 level in the rats' hearts (*n* = 7) after using nicorandil. One-way ANOVA statistical analysis was conducted for multiple comparisons of group means. The same was used to analyze the results of each experimental group. ^*∗*^*P* < 0.05, ^*∗∗*^*P* < 0.01, and ^*∗∗∗*^*P* < 0.001 compared with the CON group; ^#^*P* < 0.05, ^##^*P* < 0.01, and ^###^*P* < 0.001 compared with the EE group.

Furthermore, we measured SOD activity and MDA levels in the rats' serum. We observed downregulation of SOD activity in the EE group, while pretreatment of high-dose nicorandil and a combination of middle-dose nicorandil and EP reversed the SOD activity. In addition, compared with the CON group, MDA levels significantly increased in the EE group and significantly decreased in all pretreatment groups in comparison with the EE group (Figure 4).

### 3.5. ET-1 Level

Considering the EE model can affect endothelial cells and further induce endothelial barrier dysfunction to aggravate myocardial injury, we further measured the level of vasoconstrictor ET-1 in the rats' hearts. The ET-1 level in the myocardium significantly increased in all groups. However, compared with the EE group, pretreatment of middle and high doses of nicorandil and the combination of middle-dose nicorandil and EP significantly decreased the ET-1 level (Figure 4).

### 3.6. TNF-*α* and IL-6 Levels

**Figure 5:**
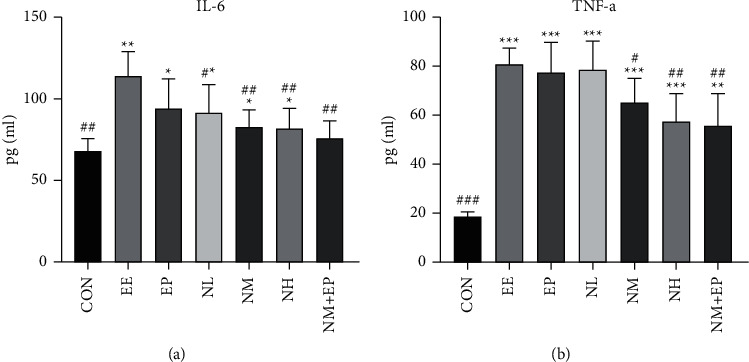
Effects on the TNF-*α* and IL-6 levels in the rats' serum (*n* = 7) after using nicorandil. One-way ANOVA statistical analysis was conducted for multiple comparisons of group means. The same was used to analyze the results of each experimental group. ^*∗*^*P* < 0.05, ^*∗∗*^*P* < 0.01, and ^*∗∗∗*^*P* < 0.001 compared with the CON group; ^#^*P* < 0.05, ^##^*P* < 0.01, and ^###^*P* < 0.001 compared with the EE group.

Inflammatory cytokines were evaluated by detecting TNF-*α* and IL-6 in the serum from all groups. The results showed an increase in TNF-*α* and IL-6 levels in the EE group compared with those of the CON group. The addition of nicorandil (middle and high doses) and the combination of middle-dose nicorandil and EP attenuated the increase of inflammation levels caused by EE (Figure 5).

### 3.7. Expression of Cardiac Troponin T and I

**Figure 6:**
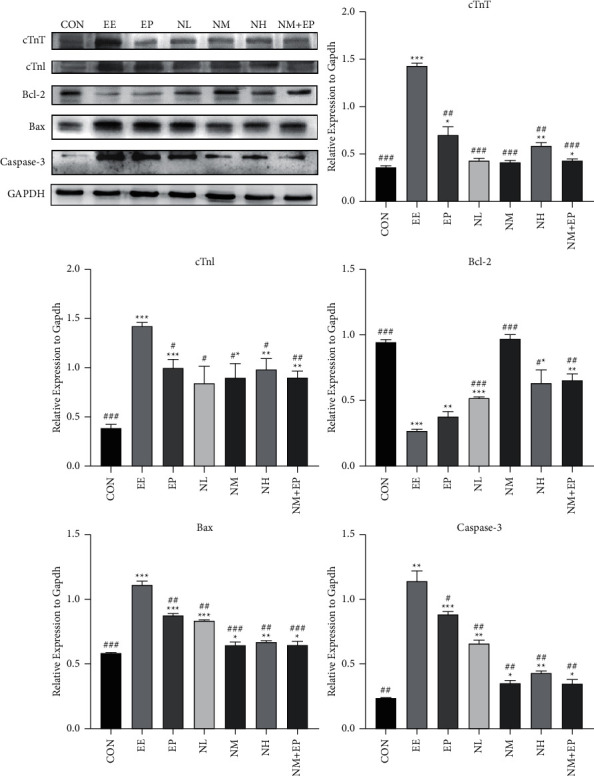
Western blotting assay of cTnT, cTnI, Bcl-2, Bax, and Caspase-3 expression. Results were shown as fold change compared with the expression of GAPDH. Data were shown as mean ± SD (*n* = 4). ^*∗*^*P* < 0.05, ^*∗∗*^*P* < 0.01, and ^*∗∗∗*^*P* < 0.001 compared with the CON group; ^#^*P* < 0.05, ^##^*P* < 0.01, and ^###^*P* < 0.001 compared with the EE group.

We further observed the expression of cardiac troponin T (cTnT) and cTnI in the heart. The western blot showed that the expression of cTnT and cTnI was significantly increased in all groups compared with that of the CON group, while pretreatment of EP and nicorandil and the combination of NM and EP decreased the expression of cTnT and cTnI in comparison with that of the EE group (Figure 6).

### 3.8. Expression of the Bcl-2 Family Proteins and Caspase-3

The Bcl-2 family of proteins, which includes Bcl-2 and Bax, is involved in the regulation of apoptosis. Caspase-3 is the executioner of apoptosis. The effects of nicorandil on the expression of Bcl-2 protein family members and caspase-3 in rat hearts were evaluated using western blotting analysis. As shown in Figure 6, EE caused a significant upregulation of Bax and caspase-3 and a downregulation of Bcl-2. Nicorandil pretreatment prevented these effects.

### 3.9. eNOS Protein Level and NO Production

**Figure 7:**
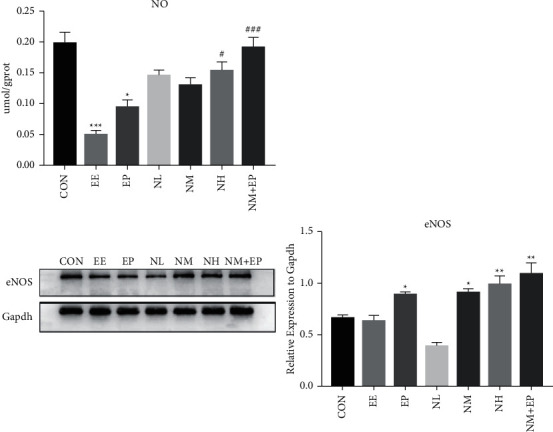
Effects on the level of eNOS protein and NO production in the rats' left ventricle myocardium. Western blotting was used to assess eNOS. Results were shown as a fold change compared with the expression of GAPDH. Data were shown as mean ± SD (*n* = 4). ^*∗*^*P* < 0.05, ^*∗∗*^*P* < 0.01, and ^*∗∗∗*^*P* < 0.001 compared with the CON group; ^#^*P* < 0.05, ^##^*P* < 0.01, and ^###^*P* < 0.001 compared with the EE group.

eNOS-mediated NO production has been known as a potent modulator of vascular smooth muscle tone and organ perfusion [14]. In addition, nicorandil itself functions as an NO donor. To further investigate the potential mechanism of the protective effects nicorandil plays in the EE model, we measured the NO production and the protein level of eNOS in the rats' hearts. Compared with the CON group, the level of NO production reduced significantly in the EE and EP groups, while pretreatment of nicorandil reversed the production of NO at NH and NM + EP in comparisons with the EE group. Western blot results showed that the level of eNOS protein decreased in the EE group and increased in the EP, NM, NH, and NM + EP groups in comparison with the CON group (Figure 7).

## 4. Discussion

Regular exercise enhances cardiac function. However, acute EE will induce heart injury such as injured cardiac ultrastructure, mitochondrial dysfunction, cardiac function decrease, and even sudden death of sports occurs [15, 16]. Nicorandil provides the medical field with a means of managing cardiac damage in patients with acute or chronic ischemic heart disease. Here, we demonstrated that pretreatment of different doses of nicorandil gavage or EP or a combination of both can protect EE hearts from acute EE-induced myocardial injury. This protection may be related to the reactive oxygen species (ROS) scavengers (SOD) and eNOS/NO signaling pathway.

Nicorandil works as a KATP channel opener and NO donor and is mainly involved in several mechanisms such as improvement of myocardial blood perfusion, reduction in preload and afterload, protection against ischemic damage, antiarrhythmic effects, prevention of calcium overload, energy-modulating actions, anti-inflammatory, antiapoptotic, and antiproliferative effects [17, 18]. However, studies mainly focused on coronary heart disease such as myocardial infarction. EE, which always happens in the athlete population with regular high-intensity training was often overlooked and has not been investigated yet. In our study, HE and electron microscope results showed that EE exposure damaged the cardiac muscle fiber and ultrastructure of the myocardium, especially the mitochondria, while pretreatment of nicorandil normalized the structure of the heart. In addition, myocardial injury biomarkers including CK, CK-MB, LDH, and cTnI increased significantly in the EE group while pretreatment of nicorandil for 4 weeks attenuated this injury according to the serum injury biomarker level (Figure 3). These results indicate the preventive role ofnicorandil gavage in the EE model. We confirmed our findings of the effects of nicorandil on cTnT and cTnI in heart tissue using Western blot analysis. Nicorandil decreased protein levels of cTnT and cTnI.

Apoptosis typically proceeds through one of two signaling cascades, known as extrinsic and intrinsic pathways, both of which converge on activating caspase-3, the executioner [19]. The results in Figure 6 show that caspase-3 expression significantly increased in the EE group compared with that in the CON group. The pretreatment with nicorandil and EP significantly decreased caspase-3 expression. The Bcl-2 and Bax protein levels are directly related to apoptosis regulation. Bax promotes cell apoptosis, whereas Bcl-2 inhibits it. Our findings demonstrate that nicorandil treatment induces an increase in Bcl-2 expression and a decrease in Bax expression. All of these results indicated the antiapoptotic effect of nicorandil in the EE model.

Nicorandil has an anti-inflammatory effect [18]. However, it is not clear how it plays a role in the EE model. We observed the level of TNF-*α* and IL-6. The results suggest that nicorandil can effectively reduce the increase of inflammatory indexes caused by EE. EE results in sustained myocardial ischemia and hypoxia. When these stimulations are beyond the limits of the body, myocardial injury occurs [1–3]. The pathological process of EE is similar to that of myocardial ischemia-reperfusion disease. Of these, oxidative stress-induced ROS accumulation is an important pathological mechanism [20], which means the broken balance between ROS production and clearance [21, 22]. In addition, ROS accumulation would further elevate the lipid level in the myocardium, and the lipid peroxidation in the myocardium would produce MDA which induces myocardial dysfunction. SOD is considered as an important myocardial ROS scavenger which can reduce the damage of ROS to the myocardium [23]. Furthermore, ROS stimulation also elevates the production of ET-1 in the myocardium and enhances the coronary constrictor response [24]. Our results showed that EE stimulation significantly lowered the protective antioxidant enzyme SOD activity and increased the MDA level in the heart, while pretreatment of high-dose nicorandil or middle-dose nicorandil plus EP significantly increased SOD activity, and all nicorandil groups and the EP group significantly decreased the level of MDA. These results indicate that the protective effects of nicorandil in the EE model may be related to the physiological processes of antioxidative stress effects. EE induced myocardial structural damage and altered myocardial damage marker levels in the circulation, which were partially reversed by nicorandil. In addition, oxidative stress leads to the accumulation of ROS products and further damage to the myocardium, while nicorandil pretreatment reduces the oxidative stress response. Finally, the pathway in which nicorandil was involved may be the eNOS/NO pathway, and nicorandil pretreatment increases the protein content of eNOS and NO production in the myocardium.

Nicorandil itself is a NO donor and may play an important role in several mechanisms during the myocardial injury process [17, 18]. NO, also known as “endothelium-derived relaxing factor (EDFR),” is a small molecular free radical that neutralizes superoxide directly to reduce oxidative stress. When myocardial injury occurs, NO bioavailability significantly reduces which further aggravates myocardial injury [25]. In our study, the NO level in myocardium was significantly reduced in the EE group while high-dose nicorandil and the combination of middle-dose nicorandil and EP significantly reversed the NO level to a normal level. These results indicate the pretreatment of nicorandil may increase the NO level by itself. Moreover, the NOS family, including neuronal NOS (nNOS), cytokine-inducible NOS (iNOS), and especially eNOS, is also crucial to NO production [26]. eNOS activation and subsequently NO production were recognized as an important mechanism during the myocardial protective process [27, 28]. Our results showed that compared with the CON group, the eNOS protein significantly increased in the EP, NM, NH, and NM + EP groups, with the NH and NM + EP groups having the highest level of eNOS compared with that of other groups. Combining the abovementioned results, the present study indicates that pretreatment of high-dose nicorandil and a combination of middle-dose nicorandil and EP may serve effective myocardial protection during the EE-induced myocardial injury process. In consideration of previous research studies focusing on the activation of eNOS, our results showed that the pretreatment of high-dose nicorandil and the combination of middle-dose nicorandil and EP may increase the protein level of eNOS. Activation of the protein still needs to be investigated in the future.

## 5. Conclusion

Taken together, our results demonstrated that pretreatment of different doses of nicorandil gavage or EP or a combination of both can protect EE hearts from acute EE-induced myocardial injury in a dose-dependent manner. This attenuation may be related to the ROS scavengers (SOD) and eNOS/NO signaling pathway.

## Data Availability

The data in the manuscript are from experiment results. More detailed supporting data can be provided upon request.

## Abbreviations

AKT: Protein kinase B
AMI: Acute myocardial infarction
CK: Creatine kinase
CK-MB: Creatine kinase isoenzymes
cTnI: Cardiac troponin I
cTnT: Cardiac troponin T
EE: Exhaustive exercise
eNOS: Endothelial nitric oxide synthase
EP: Exercise preconditioning
HE: Hematoxylin and eosin
iNOS: Inducible nitric oxide synthase
IP: Ischemic preconditioning
I/R: Ischemia/reperfusion
KATP: Adenosine triphosphate-sensitive potassium
LDH: Lactate dehydrogenase
MDA: Malondialdehyde
mTOR: Mammalian target of rapamycin
nNOS: Neuronal nitric oxide synthase
NO: Nitric oxide
PI3K: Phosphatidylinositol 3-phosphate kinase
ROS: Reactive oxygen species.
